# Intermittent use of sulphadoxine-pyrimethamine for malaria prevention: a cross-sectional study of knowledge and practices among Ugandan women attending an urban antenatal clinic

**DOI:** 10.1186/1475-2875-13-399

**Published:** 2014-10-11

**Authors:** Charles O Odongo, Ronald K Bisaso, Josaphat Byamugisha, Celestino Obua

**Affiliations:** Department of Pharmacology and Therapeutics, School of Biomedical Sciences, College of Health Sciences, Makerere University, Kampala, Uganda; Department of Pharmacology and Therapeutics, Faculty of Medicine, Gulu University, Gulu, Uganda; Department of Obstetrics and Gynaecology, School of Medicine, College of Health Sciences, Makerere University, Kampala, Uganda

**Keywords:** Malaria, IPTp, Sulphadoxine-pyrimethamine, Pregnant women, Uganda

## Abstract

**Background:**

The WHO recommends supervised administration of sulphadoxine-pyrimethamine (SP) as intermittent preventive treatment for malaria (IPTp) during pregnancy. Logistical constraints have however favoured unsupervised intake of SP-IPTp, casting doubts whether recent guidelines requiring more frequent intake can be effectively implemented. To propose strategies for enhancing compliance under limited supervision, this study sought to identify pregnant women’s knowledge and practices gaps as well as determine predictors of compliance with SP-IPTp, given under limited supervision.

**Methods:**

A cross-sectional study of 700 women used exit interviews at an urban clinic in Uganda to obtain a descriptive summary of demographic and obstetric characteristics, including knowledge, practice and experiences with SP. Predictors of compliance with SP intake instructions were explored using logistic regression.

**Results:**

Median age of respondents was 25 (IQR 22–28) and median parity was two (IQR one to three) while median number of antenatal clinic (ANC) visits was 3.0 (IQR three to four). Most women had completed primary (36%) or ordinary secondary education (25.6%) while 16.1% had not completed primary education. Awareness about SP was high (99.4%) although correct knowledge regarding its use in pregnancy was low (57%), with 15.4% thinking it was used to treat malaria and 26.7% lacking any idea about its use. Correct knowledge on SP use during pregnancy significantly predicted compliance with SP-IPTp instructions (OR 1.98, C.I. 1.12-3.55), while age, education level, parity, number of ANC visits, or history of unwanted effects with SP did not. SP was mostly accessed from hospitals (64.4%) followed by private clinics (16.9%) both for preventive and treatment purposes. SP was considered safe by most women, who were willing to take it again in future, without supervision.

**Conclusion:**

Despite high awareness, knowledge of SP as an intervention for malaria prevention in pregnancy was low. Correct knowledge on use of SP predicted compliance with SP-IPTp intake instructions. Focused malaria-related education during ANC visits may improve compliance with SP intake amidst limited supervision.

**Electronic supplementary material:**

The online version of this article (doi:10.1186/1475-2875-13-399) contains supplementary material, which is available to authorized users.

## Background

Malaria continues to be an important public health problem in Africa where it contributes significantly to adverse pregnancy outcomes [1]. Over 30 million pregnancies at risk of malaria occur in Africa each year [1]. Uganda, having the third highest fertility rate in the world [2], bears a considerable proportion of the malaria burden in pregnancy. Intermittent preventive treatment of malaria during pregnancy (IPTp), with sulphadoxine-pyrimethamine (SP) is a key component of the WHO’s strategy to mitigate the adverse impact of malaria on pregnancies in Africa [3]. Despite the wide adoption of IPTp as policy in malaria-endemic countries, there has been a global debate as to whether SP is still the most appropriate drug [4, 5]. Critical among the concerns has been the rising tide of SP resistance across Africa [6–8]. However, because the efficacy requirement for prevention is less than that for treatment of clinical malaria, technical reviews have consistently shown that SP-IPTp is still beneficial, even where prevalence of resistance is considerably high [9–11].

Alternative delivery mechanisms for IPTp aimed at reaching as many pregnant women as possible have been another area of debate [12–14]. The WHO recommends that IPTp be given through antenatal clinic (ANC) platforms to all pregnant women under the direct supervision of a health worker [3]. However, reports from various African countries suggest that this has been particularly difficult to achieve, mainly because of logistical constraints imposed by high client numbers [15–19]. As such, unsupervised intake of SP-IPTp is the reality in many settings, a scenario that requires the full cooperation of pregnant women if implementation of this policy is to succeed.

Even though multiple factors such as perceived risk-benefits, provider-client relationship, previous drug experiences among others, are known to influence compliance with oral medication [20, 21], pregnancy comes with additional challenges. For instance, physiological changes create a general aversion to oral medication [22]. This is likely to reduce compliance especially in circumstances where intake is unsupervised. Furthermore, pregnant women tend to over-estimate the risks associated with drug use during pregnancy [23]. Such fears have been particularly documented with SP in Nigeria [15] and Uganda [24]. It is likely that such considerations underlie reports that some pregnant women receive but do not take IPTp medication [15, 25]. Therefore, implementation of the revised WHO policy [26], requiring more frequent administration of SP-IPTp is likely to pose a formidable challenge under the current circumstances. If the full impact of this revision is to be realized, there is need for specific interventions aimed at improving pregnant women’s willingness to take SP under limited supervision. In order to gain insight and propose mitigation strategies against the above challenges, this study sought to identify knowledge and practice gaps associated with SP-IPTp among pregnant women in Uganda. Specifically, the study explored a range of factors so as to determine predictors of compliance with SP-IPTp under the current circumstances. Lastly, the study describes pregnant women’s perceived adverse experiences with SP and examines their willingness to take it again under limited supervision.

## Methods

### Study design

This was a cross-sectional study encompassing both descriptive and analytical aspects. Data for this study were collected as part of a wider cross-sectional study whose objectives included exploration of pregnant women’s knowledge and experiences with malaria during pregnancy, determination of their anti-malarial drug sources and use patterns during pregnancy, exploration of their knowledge and practices regarding SP-IPTp and description of their previous experiences with SP-IPTp. Exit interviews were used to collect data from pregnant women attending antenatal care at an urban hospital in Uganda. Even though data for the above objectives were all collected concurrently, using the same tool and from the same sample population, this paper addresses only the last two objectives.

### Study site and study population

Mulago National Referral and Teaching Hospital is a 1,500-bed complex located in Kampala, the capital and commercial centre of Uganda. It offers a wide range of specialized care and runs free outpatient clinics on weekdays. Most outpatients come from the urban and peri-urban communities of Kampala and the neighbouring districts of Mukono and Wakiso. These areas are mainly inhabited by the ethnic Bantu, particularly the Baganda. Over time however, other Ugandan ethnic communities have settled here constituting a significant minority. Most outpatients visiting the hospital tend to be low- and middle-income earners who prefer to utilize the free services offered at the hospital. Records show that over 1,000 outpatients are seen daily, with approximately 20% of these being pregnant women seeking antenatal care at the hospital’s two ANC clinics. All women initially present to the general ANC located at Old Mulago. This clinic is run by a team of experienced midwives and offers a standard package of routine antenatal care services. If the opinion of an obstetrician is required or the pregnancy is considered of a high-risk nature, women are referred to the New Mulago ANC. Since all women are initially seen at the general ANC clinic, only attendees from this clinic were approached for the study. The current government policy on IPTp is that all HIV-negative women should get at least two doses of SP-IPTp during each pregnancy, while HIV-positive women are initiated on daily co-trimoxazole prophylaxis instead of SP-IPTp. For the management of clinical malaria during pregnancy, the policy recommends use of quinine in the first trimester of gestation and ACT or quinine in second and third trimesters [27].

### Antenatal clinic setting

The general ANC clinic runs from 08:00 to 16:00 hours on all weekdays and is attended by an average of ninety pregnant women on each day. All services (including medications) are offered free to all patients. Daily activities begin with an hour-long group talk offered to all women attending on that day, regardless of age, education level, parity, number of previous visits or gestational age category. This session is conducted by the clinic in-charge who ensures that all women visiting the clinic attend. Issues covered include pregnancy-related health education, counselling on pregnancy and emergency preparedness, nutrition, hygiene, birth plan, post-partum care, breast feeding, prevention of sexually transmitted infections and family planning. During this session, women are encouraged to ask questions. After the group talk, women are directed into private consultation rooms where they are offered additional services which include a standard antenatal examination, voluntary counselling and testing for HIV and syphilis, and tetanus immunization. Depending on the case, a woman may be referred or sent to the laboratory or ultrasound room for further investigation. For those with normal pregnancies, prescriptions for SP-IPTp, haematinics or anti-helminthic tablets may be given, whichever is due. Prescriptions for other minor intercurrent illnesses may also be given. The date for their next visit is given and women are discharged via the clinic pharmacy where their medication is dispensed.

### Sample size determination

A minimum sample size of 385 was estimated to answer the current objectives using the formula of Cochran [28] based on the following parameter assumptions; a 5% level of precision (sampling error) and a standard normal deviate (Z_α_) of 1.96 (for a confidence level of 95%). A maximum degree of variability (0.5) in compliance with IPTp intake instructions was assumed as there was paucity of data on compliance with SP-IPTp from this population, or any other similar population. As this study was part of a larger study on malaria and anti-malarial drug use among pregnant women, a final sample size of 700 computed to cover the other objectives was used.

### Research instrument

A structured questionnaire, mainly consisting of close-ended questions was developed based on themes contained in the malaria in pregnancy treatment and prevention models proposed by Ribera et al. [29] (see Additional file 1). The questionnaire included questions on sociodemographic and obstetric characteristics relevant to study objectives. Areas of inquiry identifying SP knowledge gaps included whether women had ever heard about SP, whether they knew about its role as given during pregnancy and whether they recalled the health workers’ dosing instructions. Areas of inquiry identifying practice gaps included history of SP use during pregnancy, the source of SP, reasons for which SP was used (if positive) during pregnancy, number of tablets received, and number of tablets actually taken. Lastly, a section on unwanted effects that women attributed to SP use, as well as their willingness to use SP again in future was included. Prior to actual data collection, the questionnaire was pre-tested on 50 pregnant women from the same clinic. This exercise allowed the research team to standardize questions and cater for all possible responses so as to ensure internal validity. In addition, potential bottlenecks to smooth conduct of both clinic and study activities were identified and streamlined.

### Recruitment and data collection

Recruitment and data collection for the entire study was done over three weeks between 5^th^ and 23rd August 2013. Pregnant women leaving the consultation rooms were consecutively approached and invited to take part in the study. This strategy served to exclude HIV-positive women who upon diagnosis, are normally referred to the New Mulago ANC clinic (located on another building). All other potential participants regardless of gestational age were eligible for recruitment. Information about the study was explained to each woman before they gave oral consent to participate. Three female students of nursing were trained and assisted in this exercise. On each clinic day, data collection begun at 09:00 and ended at 13:00 hours. Each research assistant administered the questionnaire to an average of 15 pregnant women per day, with each interview lasting between ten to fifteen minutes. The interviewers were free to ask any other specific questions in order to satisfy themselves that SP was not being confused with other routine ANC medications. Communication was either in English or a local vernacular language (whichever was convenient for individual women). Whilst in the clinic, one of the investigators scrutinized all completed questionnaires to ensure correctness and completeness of records before they were filed.

### Data management and statistical analysis

Data were coded, double-entered and validated in *EpiData*^®^ [30] from where it was exported to R statistical computing software [31] for analysis. Descriptive statistics were used to summarize sociodemographic, obstetric, as well as knowledge, practice and SP experience data. Compliance with SP-IPTp intake instructions was defined as participants who reported receiving three tablets of SP and took all three tablets at once. This was the main outcome variable in this study. Other variables such as participant age, education level, parity, number of ANC visits in most recent pregnancy and correct knowledge on the role of SP (as used during pregnancy) were investigated as predictors of compliance with SP intake instructions. As regards knowledge on the role of SP, one was considered to have correct knowledge if they said SP was used to prevent malaria. Thus, for purposes of analysis, there were only two categories for this variable. Multicollinearity was examined between all variables by calculating the variable inflation factor (VIF) for each predictor variable. Binomial logistic regression was then used to investigate each, beginning with univariate analysis. Odds ratios were computed for each variable with statistical significance set at p ≤0.05 at the 95% confidence level. In order to avoid confounding bias in effect estimates arising when bivariate selection is used for inclusion of variables in multivariate models [32], all variables regardless of significance, were included in the full multivariate model. The robustness of the final model was validated by non-parametric bootstrapping, with 1,000 new datasets randomly generated from the original dataset.

### Ethical considerations

In order to avoid the possible negative influence of written consent on rapport between interviewer and respondent, women were required to give only oral informed consent. In any case, the study was also deemed to pose minimal risks to participants. Confidentiality was maintained by ensuring one-on-one interviews in private, well-lit rooms. Numeric identification codes were used in order to conceal individual identities and all records were securely handled by study staff. The study protocol was reviewed and approved by the Mulago Hospital Research and Ethics Committee (ref MREC number 397) and permission to conduct the study was granted by the Uganda National Council for Science and Technology (UNCST).

## Results

### General characteristics of pregnant women surveyed

All women approached for the study consented to participate. The median age (and IQR) was 25.0 (22–28) years with a minimum and maximum of 16 and 43 years, respectively. Median gestational age (and IQR) was 24.0 (20–32) weeks with a minimum and maximum of four and 36 weeks, respectively. Other characteristics of the women surveyed are presented in Table 1.

**Table 1:** **General characteristics of pregnant women surveyed (n = 700)**

Characteristic	Number of respondents	Proportion (%)
**Parity** (number of previous pregnancies ≥28 weeks)		
First pregnancy	148	21.1
Second pregnancy	173	24.7
Third or more pregnancies	379	54.2
**Number of ANC visits in last pregnancy** (n = 552)*		
None	32	5.8
One or two visits	90	16.3
Three visits	161	29.2
Four or more visits	269	48.7
**Education level completed**		
None or never completed primary	113	16.1
Primary education	252	36.0
Ordinary level education	179	25.6
Advanced level education	87	12.4
Tertiary education	69	9.9
**Knowledge on SP-IPTp use during pregnancy**		
Prevent malaria in mother (or unborn baby)	399	57.0
Treatment of malaria	109	15.4
Do not know	187	26.7
Other response	6	0.9
Experienced SP-IPTp unwanted effects (n = 629)**		
None	432	68.7
Mild (headache/dizziness, nausea/vomiting, fever)	105	16.7
Severe (skin reaction, abdominal pain, general weakness)	92	14.6

*Data excludes primigravidae, **data from only previous users of SP-IPTp.

### Knowledge associated with SP use during pregnancy

Nearly all respondents (99.4%) had heard about SP prior to the interview. Regarding knowledge on the role of SP as used during pregnancy, 57% mentioned prevention of malaria in mother or unborn baby while 15.4% thought it was used to treat malaria. About 26% and 0.9% respectively, did not know its indication or cited other reasons not related to malaria (Table 1). Regarding knowledge on the dosing of SP as per health workers’ instructions, 86.5% of respondents mentioned the correct dosing instructions i.e. three tablets all taken at once.

### Practices associated with SP use during pregnancy

Most respondents (89.9%) had used SP during pregnancy with 63.9% having used it in their current pregnancy. Most respondents (64.4%) had accessed SP from a hospital followed by a private clinic (16.9%) or other public health facility (10.2%). When asked to recall why they had used SP during pregnancy, 54.1% mentioned that it was offered on a routine ANC visit about which they had no particular complaint (or symptom) related to malaria. Twenty-six per cent reported receiving SP because they had complained of malaria symptoms while 11.3% received it on separate occasions both as routine ANC medication as well as when they had confirmed episodes of malaria during pregnancy. Approximately 9% could not recall why they were given SP during pregnancy. When asked to mention the number of SP tablets they had received along with the health workers’ dosing instructions, 86.5% reported that they were given three tablets with instructions to take all at once. This same proportion of women reported using SP in the manner instructed thus meeting the criteria that defined compliance in this study. Other respondents 4.6 and 2.5% reported receiving more than three or less than three tablets, respectively, to be taken at various times. Forty women (6.4%) could not recall the number of tablets received.

### Predictors of compliance with SP-IPTp intake instructions

All predictor variables had VIF values less than 1.86 (R^2^ < 0.46) suggesting limited collinearity between variables. This allowed for all to be independently entered into the multivariate model without fear of loss of precision in effect estimates. Logistic regression showed that participant age, education level, number of ANC visits in previous pregnancy and correct knowledge on SP-IPTp were significantly associated with compliance with SP intake instructions when independently investigated (Table 2). However, in the multivariate model, only correct knowledge on SP use remained statistically significant with effect estimates showing that it offered approximately two-fold increase in compliance with SP intake (OR 1.98, C.I: 1.12-3.55, Table 2). Results of non-parametric bootstrap analysis showed minimal bias of less than 10% for all model estimates. All estimates also lay within the bias-corrected 95% confidence intervals (Table 3).

**Table 2:** **Model outputs for predictors of compliance with instructions for SP-IPTp intake**

Predictor variables	Unadjusted OR	95% C.I	Adjusted OR	95% C.I
Participant age	1.063	1.007-1.124	1.054	0.974-1.144
Education level	1.268	1.043-1.549	1.275	0.992-1.655
Parity*	1.089	0.937-1.277	1.051	0.812-1.375
Number of ANC visits	1.201	0.999-1.445	1.112	0.918-1.348
Correct knowledge on SP-IPTp use	2.492	1.526-4.120	1.981	1.118-3.551
Experience of SP unwanted effects	1.477	0.859-2.651	1.222	0.650-2.418

Hosmer and Lemeshow goodness of fit (GOF) test: χ^2^ = 4.8907, df =8, p-value =0.7692.

*Parity = Number of previous pregnancies >28 weeks, OR = Odds ratio, CI = Confidence interval.

**Table 3:** **Model validation results from bootstrap analysis using 1000 randomly generated datasets**

	Original log odds	Bias (%)	Std. error	Bias corrected and accelerated 95% CI
Intercept	–0.998	–6.561	1.059	(–3.119 – 0.950)
Participant age	0.052	0.265	0.048	(–0.041 – 0.141)
Education level	0.243	0.871	0.132	(–0.022 – 0.494)
Parity	0.049	0.529	0.141	(–0.229 – 0.333)
No. of ANC visits	0.106	0.035	0.101	(–0.108 – 0.293)
Correct knowledge on SP	0.683	0.748	0.303	(0.067 – 1.278)
Experience of SP unwanted effects	0.201	3.397	0.363	(–0.506 – 0.881)

### Perceived unwanted effects of SP-IPTp and willingness to take it again

Overall, 68.7% of pregnant women felt that SP was safe because they did not experience any bad effects following intake. However, up to 31.3% experienced some form of unwanted effect, which they attributed to SP. The most common effects mentioned were nausea and vomiting (7.3%) and general body weakness (7.0%). Other effects mentioned were headache or dizziness (5.9%) and abdominal pain or diarrhoea (1.4%). Few participants (5.9%) reported more than one unwanted effect. Occurrence of allergic-type reactions, such as skin eruptions or fever-like symptoms, were reported by 0.3 and 0.9% of women, respectively. When asked whether they would take SP again given their previous experiences, most women (92.7%) were willing to take it again. Among the few (n = 57) who responded negatively, forty seven cited fear of injury to themselves while ten cited fear of injury to their unborn baby.

## Discussion

This study sought to identify knowledge and practice gaps associated with SP-IPTp among an urban population of pregnant women attending ANC in Uganda. Despite high awareness and access to SP during pregnancy, correct knowledge about its use in pregnancy was less than adequate. A high proportion of women (43%) either did not know the role of SP or thought it was still used for malaria treatment. This is worrisome coming from a group of pregnant women who attend ANC, because it raises doubt whether these women can willingly take SP (without supervision) in the absence of malaria symptoms.

Because antenatal care platforms are supposed to provide access to a wide range of pregnancy friendly interventions, optimum ANC attendance is probably the most important decision pregnant women and their partners make. ANC attendance in this population was suboptimal with less than half of women making the minimum recommended four visits in their most recent pregnancy. Previous studies in Uganda have reported similarly low figures: 34% in Jinja [19] and 37.1% in Luweero [33] districts. These figures however remain higher than the national average of 25% as per the most recent Demographic and Health Survey report [34]. Across sub-Saharan Africa, wide variation in ANC attendance patterns has been noted. A recent review on this subject estimated that 71% of women attend ANC at least once, with only 44% making four or more visits [35]. Reasons for non-ANC attendance have been identified in several studies [36, 37] and include issues relating to ignorance, access (availability, costs), concerns about religious or cultural violations, and the perception of having a normal pregnancy (especially common among multiparous women). In some instances, non-ANC attendance was attributed to a perceived lack of enforcement by relevant local authorities. Studies also show that women who make fewer ANC visits have the least chance of accessing pregnancy friendly interventions [37, 38]. Moreover, such few attendances tend to occur later in pregnancy, thus particularly affecting preventive interventions like malaria-related health education which is normally begun in early pregnancy. Lack of knowledge about when to start ANC visits has been identified as the most critical reason associated with late index ANC attendance at Mulago hospital [39] and this may account for some of the SP knowledge gaps identified in this study.

Over one third of pregnant women received SP following presentation with malaria symptoms. This is worrisome as it suggests that some health workers continue to prescribe (or dispense) SP for treatment of symptomatic malaria among pregnant women. SP no longer has the efficacy required for treating clinical malaria. Its use instead of quinine or ACT, therefore, increases the risk of adverse pregnancy outcomes such as abortions due to ineffectively treated malaria. These findings are not entirely new in Uganda where recent studies from Jinja [40] and Mukono [41] districts have reported a 37 and 48% use of SP among febrile pregnant women, respectively. Until now, such rampant misuse of SP was thought to be unique to rural populations. However, this report suggests that the pattern may not be different among urban women. Elsewhere, a recent study in Nigeria revealed that chloroquine and SP were the most common anti-malarial medicines used among rural pregnant women [16]. Considering that hospitals, followed by private clinics, were the most common sources of SP in this study, these findings indicate a high degree of non-adherence to treatment guidelines among health workers. This trend requires an urgent check as it carries serious implications for the well-being of pregnancies afflicted by malaria in Uganda.

Pregnant women reported a high degree of compliance with SP instructions (86.5%) when received as IPTp. Similarly, high compliance rates (77.4%) were reported by Akinleye et al. among rural Nigerian women [15]. The high compliance rate in this study and the fact that nearly all women were willing to take SP again in future, suggests that SP is perceived as a relatively safe drug. This is contrary to earlier reports in which SP was perceived as 'too strong’, thereby likely to weaken pregnant women and cause abortions or foetal abnormalities [24]. These findings are in line with earlier suggestions by Sangare et al. that when offered SP-IPTp, pregnant women will accept and use it [19]. In this study, correct knowledge on SP-IPTp was found to be a significant predictor of compliance with intake instructions. This is consistent with findings from one Nigerian study that suggested that knowledge about malaria prophylaxis in pregnancy predicted IPTp utilization among rural women [16]. Findings from the two studies underscore the notion that awareness of the health benefits of an intervention will promote its uptake, at least during pregnancy. The importance of knowledge and awareness campaigns aimed at promoting positive health practices was therefore highlighted by this study.

It was reasonable to assume that pregnancy presented an opportunity to learn about SP-IPTp or other pregnancy-related health information. This would be especially true among women who attended ANC because of the increased likelihood to interact with appropriate information sources. Since the majority of women in this study had experienced at least one previous pregnancy, for which a median of three ANC visits were made, it was anticipated that number of ANC visits (in a previous pregnancy) would positively predict reported compliance with IPTp instructions. This was however not true and may point towards a deficiency in malaria-related health education as normally given during ANC visits. Alternatively, malaria-related health messages (if any) presented during ANC visits may be inappropriately tailored to pregnant women’s needs. In addition, this observation may be a manifestation of late ANC attendance as earlier observed in this discussion.

This report may be considered unique in attempting to present actual users’ experiences and perceptions on SP safety, in contrast to most studies that have used provider perspectives. Overall, its findings are consistent with a previous review upholding the safety of SP in pregnancy [42]. Pregnant women in this population may therefore have a low risk perception towards SP considering the high compliance rate and willingness to use it again in future. Cases of non-compliance with SP intake (if any) are likely to arise due to factors such as physiological aversion to oral medication or a perceived lack of benefit (from SP) owing to limited knowledge as elaborated above.

A key limitation of this study was the exclusive reliance on respondents’ verbal reports as a measure of outcome variable (i.e., compliance with SP-IPTp intake instructions). This was subject to social desirability bias as respondents’ answers may have reflected what they believed the interviewer would find acceptable, not necessarily the fact of SP intake. As such, it is possible that some level of inaccuracy in outcome measurement occurred. Even though this was anticipated, little could be done to control for it within a cross-sectional study design. Furthermore, these findings may not be generalizable to all Ugandan women, particularly those who do not attend ANC. However, such persons cannot access SP-IPTp within the current delivery platform, hence their views are of limited consequence on the current implementation challenges. However, the robustness shown by bootstrap analysis provides a basis for generalization of study findings to ANC-attending pregnant women living in both urban and non-urban settings within Uganda. These findings may also apply elsewhere in developing countries with similar sociodemographic profiles and health system challenges. Lastly, the use of exit interviews precluded the possibility of obtaining greater insights into SP-IPTp knowledge and practices as this would have required the addition of focus group discussions. Nevertheless, the current study provides useful insights into important knowledge and practice gaps associated with SP use during pregnancy.

## Conclusion

Despite high awareness of SP, knowledge on the rationale of its use during pregnancy was less than adequate. Correct knowledge on the role of SP during pregnancy predicted compliance with SP-IPTp intake instructions. SP was perceived as a relatively safe drug and women were willing to take it again without supervision. For as long as unsupervised intake of SP-IPTp remains routine, providers should endeavour to inform and educate pregnant women on the rationale of this intervention. Information should emphasize the importance and benefits of compliance with SP-IPTp.

## Electronic supplementary material

Additional file 1: **Malaria and anti-malarial drug use patterns among pregnant women attending Mulago Referral Hospital, Kampala.** (DOC 40 KB)

## Abbreviations

ACT: Artemisinin-based combination therapies
ANC: Antenatal care
ANC: Antenatal clinic
IPTp: Intermittent preventive treatment of malaria during pregnancy
SP: Sulphadoxine-pyrimethamine
UNCST: Uganda National Council for Science and Technology
VIF: Variable inflation factor
WHO: World Health Organization.
