# Supplementary material for: Intermittent use of sulphadoxine-pyrimethamine for malaria prevention: a cross-sectional study of knowledge and practices among Ugandan women attending an urban antenatal clinic
Source: Malar J. 2014 Oct 11;13:399. doi: 10.1186/1475-2875-13-399 (PMC4210552; doi:10.1186/1475-2875-13-399)
Supplement: Supplementary file 1 — Additional file 1: Malaria and anti-malarial drug use patterns among pregnant women attending Mulago Referral Hospital, Kampala. (DOC 40 KB) [file 12936_2014_3574_MOESM1_ESM.doc]

**MALARIA AND ANTIMALARIAL DRUG USE PATTERNS AMONG PREGNANT WOMEN ATTENDING MULAGO REFERRAL HOSPITAL, KAMPALA**

**Investigator: Dr. Odongo Charles**

**Objectives:**

1. **To explore pregnant women’s knowledge and experiences with malaria during pregnancy**
2. **To determine antimalarial drug sources and use patterns among pregnant women**
3. **To explore pregnant women’s knowledge on the use of SP (Fansidar) during pregnancy**
4. **To describe pregnant women’s previous experiences with SP (Fansidar) as IPTp**

**PART I: Participant demography and obstetric history:**

STUDY / SERIAL NO: …………………..

1. Participant Age: …………………… and Gestational Age: ……………….…
2. Highest level of education attained:

(i) None (never went to school) (ii) Never completed primary education

(iii) Completed primary education (iv) Completed O’ level (S. 4)

(v) Completed A’ level (S. 6) (vi) Completed tertiary education

1. No. of previous pregnancies carried (with or without completion): ………..…
2. In your last pregnancy, how many times did you attend antenatal visits? ………….

**PART II: Knowledge and experience with malaria and malaria during pregnancy**

1. Have you ever suffered from malaria fever? (tick as appropriate) YES NO
2. How did you feel? (tick all that are mentioned)
3. Headache b) Fever c) Backache d) Muscle aches e) General body weakness

f) Abdominal pain g) Yellow eyes h) Nausea/vomiting i) Sweating j) Joint pains

k) Others (specify) …………………………………………………………………….

1. If you have ever suffered from malaria when not pregnant, what medicine did you use to treat that episode?

……………………………………………………………………………………………….

1. If you have ever suffered from malaria in this pregnancy, what medicine did you use?

…………………………………………………………………..

1. Where did you access the malaria medicine you used when pregnant?
2. Drug shop b) Nearby clinic c) Pharmacy d) Public health center d) Hospital

**PART III: Knowledge and experiences with Sulfadoxine-Pyrimethamine (Fansidar)**

1. Have you heard of the medicine called Fansidar? (tick as appropriate) YES NO
2. Have you ever received (or used) Fansidar during pregnancy? YES NO
3. From which health unit did you receive Fansidar while pregnant? (tick all that apply)

a) Drug shop b) Private clinic c) Pharmacy d) Public health center e) Hospital

1. Do you remember why you were given Fansidar during pregnancy?

a) Had malaria /feeling unwell b) routine antenatal visit c) don’t know/remember

1. How many tablets of Fansidar did you receive and how were you instructed take them? ……………………………………………………………………………………………………
2. How did you actually take Fansidar during pregnancy? ……………………………………………………………………………………………………
3. Did you have any bad effects after taking Fansidar while pregnant? (tick all mentioned) (i) Headache/Dizziness, ii) Abdominal pain iii) Nausea/vomiting iv) Fever v) General weakness vi) Skin reaction vii) other …………………………………………
4. Have you used Fansidar in the current pregnancy? YES NO
5. What do you think is the role of Fansidar when given during pregnancy?
6. Prevent malaria (ii) treat malaria (iii) don’t know

(iv) Other (specify) ………………………………………………………………………

1. From past experience, would you use Fansidar again during pregnancy? YES NO
2. If no, why?.......................................................................................................
